# Strategic behavior of large language models and the role of game structure versus contextual framing

**DOI:** 10.1038/s41598-024-69032-z

**Published:** 2024-08-09

**Authors:** Nunzio Lorè, Babak Heydari

**Affiliations:** 1 Multi-Agent Intelligent Complex Systems (MAGICS) Lab, Network Science Institute, Northeastern University, Boston, MA USA; 2 Multi-Agent Intelligent Complex Systems (MAGICS) Lab, College of Engineering and Network Science Institute, Northeastern University, Boston, MA USA

**Keywords:** Computational science, Psychology and behaviour

## Abstract

This paper investigates the strategic behavior of large language models (LLMs) across various game-theoretic settings, scrutinizing the interplay between game structure and contextual framing in decision-making. We focus our analysis on three advanced LLMs—GPT-3.5, GPT-4, and LLaMa-2—and how they navigate both the intrinsic aspects of different games and the nuances of their surrounding contexts. Our results highlight discernible patterns in each model’s strategic approach. GPT-3.5 shows significant sensitivity to context but lags in its capacity for abstract strategic decision making. Conversely, both GPT-4 and LLaMa-2 demonstrate a more balanced sensitivity to game structures and contexts, albeit with crucial differences. Specifically, GPT-4 prioritizes the internal mechanics of the game over its contextual backdrop but does so with only a coarse differentiation among game types. In contrast, LLaMa-2 reflects a more granular understanding of individual game structures, while also giving due weight to contextual elements. This suggests that LLaMa-2 is better equipped to navigate the subtleties of different strategic scenarios while also incorporating context into its decision-making, whereas GPT-4 adopts a more generalized, structure-centric strategy.

## Introduction

Large language models (LLMs) such as GPT from OpenAI and LLaMa-2 from Meta have garnered significant attention for their ability to perform a range of human-like tasks that extend far beyond simple conversation. Some argue that these models may serve as an intermediate step toward artificial general intelligence (AGI)^1^. Recent advancements have shown GPT-4 passing the bar exam^2^ and GPT-3 solving complex mathematical problems^3^. Despite these achievements, these models exhibit limitations, notably in tasks like network structure recognition^4^.

Social and behavioral science research on large language models (LLMs), including GPT and LLaMa-2, is divided into two principal streams: one that explores human-like cognitive capabilities such as reasoning and theory of mind^5–7^, and another that evaluates performance in comparison to human skills across a variety of tasks^8–10^. In the field of economics, the emphasis is predominantly on performance evaluation, exploring applications like market research and sentiment analysis^11–13^. This dual focus coalesces in social science research, where LLMs are being tested for both their cognitive reasoning skills and performance outcomes within the intricate framework of social dilemmas and game theory^14–17^. Research in this space is motivated by several distinct goals, chief among them the possibility of using these algorithms as substitutes or surrogates of human subjects in experimental settings^18–23^. In a market that is increasingly hungry for algorithmic assistants that can aid human decision-making^24,25^, LLMs could prove to be an invaluable asset if their capabilities for strategic reasoning were to be demonstrated. Beyond industrial and academic applications, an active and ongoing area of research involves military simulations and war games with large language models^26–28^. Strategic considerations seamlessly extend from the realm of classic game theory into applications for national defense and security, underscoring the importance of understanding the extent to which these algorithms can adeptly navigate interactive non-cooperative scenarios.

Moreover, investigating the strategic behavior of large language models is intricately linked to studying their theory of mind (ToM) capabilities^29–32^. Strategic decision-making often requires understanding and predicting the actions, beliefs, and intentions of others, which is a core aspect of ToM. By evaluating how LLMs, such as GPT-4 and LLaMa-2, adjust their strategies based on contextual information and social cues, researchers can gain insights into the models’ ability to simulate human-like ToM.

Existing studies indicate that LLMs can mimic human behavior to some extent^33,34^, yet their aptitude for strategic decision-making in game-theoretic contexts is still an area for exploration^35–38^. Beyond the structural elements of a game, the contextual framing can significantly affect decision-making processes. Prior research on human behavior has underlined the powerful role of context in shaping strategic choices; for example, the framing of a game as a Wall Street venture versus a community endeavor led to divergent decisions^39–42^. As a result, our study aims to go beyond assessing the fundamental strategic capabilities of LLMs, also considering the influence of game structure and contextual framing on their decision-making.

To disentangle the complexities of strategic decision-making in LLMs, we conduct a series of game-theoretic simulations on three distinct models: GPT-3.5, GPT-4, and LLaMa-2. We focus on social dilemmas, games in which players may either cooperate for collective benefit or defect for individual gain. Starting from the well-known Prisoner’s Dilemma, we expand our study to include other two-player games such as the Stag Hunt, Snowdrift, and Prisoner’s Delight (aka Harmony Game). Besides examining these games, we introduce five different contexts—ranging from business and diplomatic discussions to casual interactions between friends—to evaluate how contextual framing influences strategic choices. Our primary research question is to determine the relative significance of game structure versus contextual framing in shaping the behavior of these models.

Our study delineates the nuanced responses of different Large Language Models (LLMs) to strategic scenarios, revealing significant variations in their strategic reasoning and contextual adaptability. GPT-3.5 exhibits heightened sensitivity to contextual framing but lacks proficiency in abstract strategic considerations, particularly in employing best response strategies. In contrast, GPT-4 and LLaMa-2 demonstrate a more balanced approach, integrating both game structure and context in their decision-making processes. Notably, the impact of context is more pronounced in scenarios framed as games among friends, where the game structure becomes secondary.

When comparing GPT-4 and LLaMa-2, GPT-4 shows a greater emphasis on game structure over context but fails to differentiate between distinct game types, categorizing them into binary buckets for “high” and “low” social dilemma. LLaMa-2, however, displays a more nuanced understanding of various game structures, incorporating both strategic and contextual factors into its analysis. This model is adept at navigating different strategic scenarios, suggesting a more sophisticated approach to integrating context with game-theoretic principles. Additionally, anecdotal evidence highlights that GPT-3.5 frequently errs in strategic advice, while GPT-4, despite its structural focus, occasionally mischaracterizes game scenarios. LLaMa-2, with its explicit game-theoretic foundation and contextual layering, offers a more comprehensive and precise strategic analysis.

The rest of this paper is structured as follows. In "Results" section we present our results across algorithms and treatments, and in "Discussion" section we discusstheir implications. We present our methods and model choices in "Methods" Section

## Results

**Figure 1:**
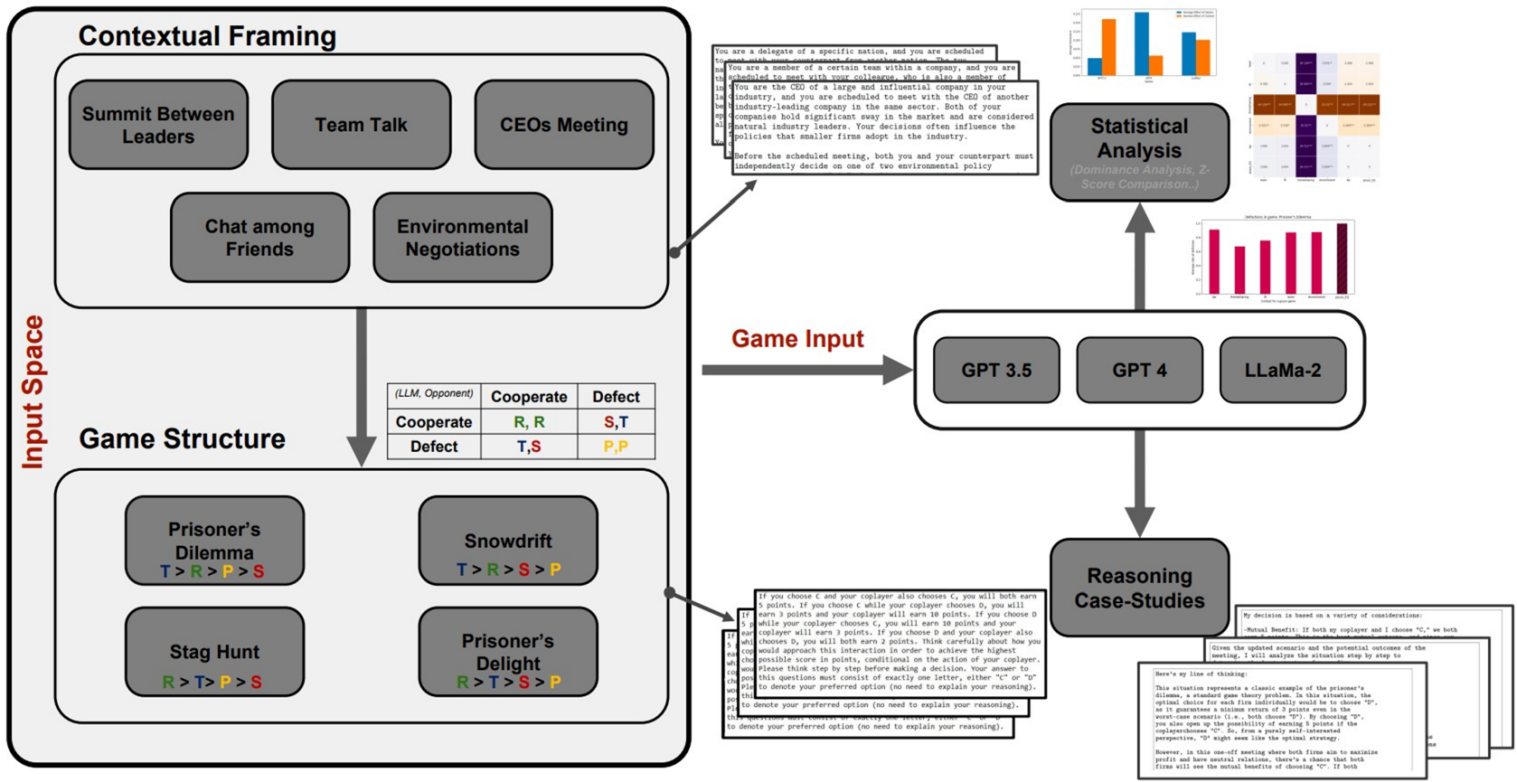
A schematic explanation of our data collecting process. A combination of a contextual prompt and a game prompt is fed into one of the LLM we examine in this paper, namely GPT-3.5, GPT-4, and LLaMa-2. Each combination creates a unique scenario, and for each scenario we collect 300 initializations. The data for all scenarios played by each algorithm is then aggregated and used for our statistical analysis, while the motivations provided are scrutinized in our Reasoning Exploration section.

Figure 1 shows the schematic workflow of this research and the process through which we generate our results. To each game, we combine a context, a term we use to indicate the social environment in which the interaction described by the model takes place. We run 300 initializations per LLM for each of the 20 possible unique combinations of context and game, before aggregating the results in order to conduct our statistical analysis.

**Figure 2:**
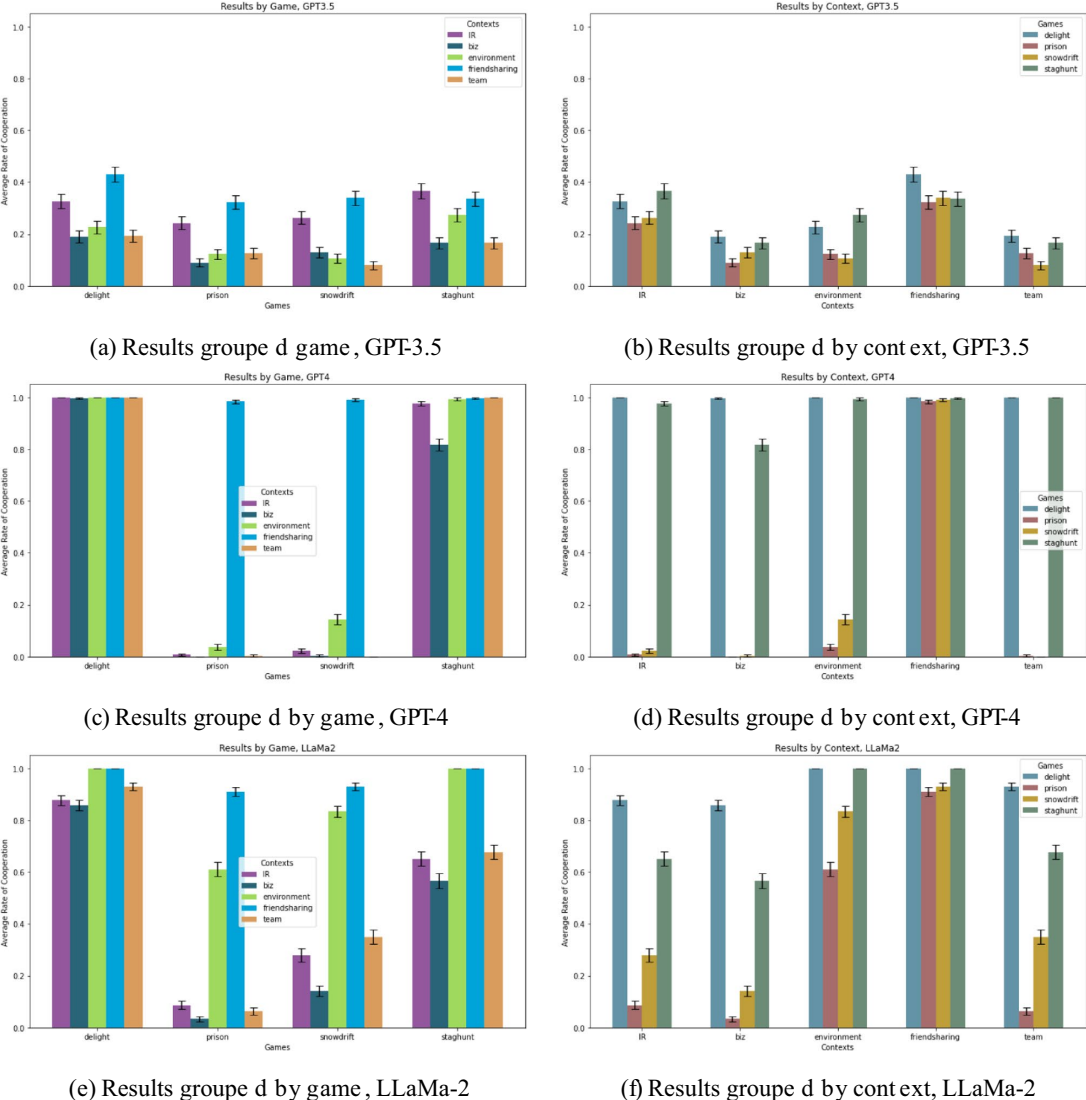
Summary of our findings, displayed using bar charts and outcomes grouped either by game or by context. On the *y* axis we display the average propensity to cooperate in a given game and under a given context, with standard error bars. (**a**) and (**b**) Refer to our experiments using GPT-3.5, and anticipate one of our key findings: context matters more than game in determining the choice of action for this algorithm. (**c**) and (**d**) Instead show how the opposite is true for GPT-4: almost all contexts are more or less playing the same strategy, that of cooperating in two of the four games and defecting in the remaining two. Finally, (**e**) and (**f**) present our results for LLaMa-2, whose choice of action clearly depends both on context and on the structure of the game.

Figure 2 displays an overview of our results for all three LLMs. To better clarify the role of game structure vs. framing context, results are aggregated at different levels: we group the observations at the game level on the left at the context level on the right, and each row represents a different LLM. A few things appear immediately clear when visually inspecting the figure. First, GPT-3.5 tends not to cooperate regardless of game or context. Second, GPT-4’s choice of actions is almost perfectly bimodal, with either full cooperation or full defection. Finally, LLaMa-2’s behavior approximates that of GPT-4 to a certain extent, but with a wider degree of variation between response both across games and across contexts. A more detailed view of strategic choice for each game, context and LLM is presented in SI B.I.

**Figure 3:**
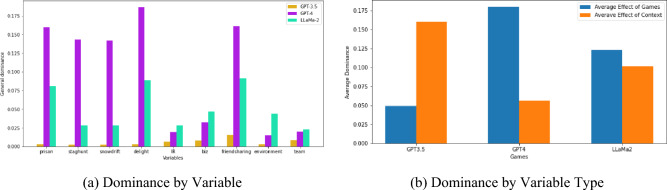
General dominance statistic for each variable, grouped by LLM. Variables with a higher dominance have a larger impact on a LLM’s final decision. The details for how these statistics are calculated can be found in the Methods section. (**a**) reports individual dominance across variables for each LLM, whereas (**b**) reports average dominance grouped by variable type (context or game) for each LLM.

To further corroborate and substantiate our findings, we turn to dominance analysis using STATA (details provided in the Methods section)^43^. We run a logit regression for each LLM encoding each game and each context as a dummy variable, and then we use dominance analysis to identify which dummies have the largest impact on the dependant variable. The output is presented in Fig. 3a. We notice that Friends’ Chat (“friendsharing”) dominance statistic across all algorithms is persistently high, and indeed by analyzing Fig. 2 it appears immediately clear that this context is consistently associated with higher rates of cooperation regardless of game or LLM. For GPT-3.5, contexts represent the five most important variables, while among games those with a sole justifiable action are associated to larger dominance statistics compared to those with multiple justifiable actions. This suggests that GPT-3.5 might have a tendency to put weight on context first and on game structure last, with a slight bias for “simpler” games. For GPT-4, on the other hand, the ranking is almost perfectly inverted with games being the regressors with the highest dominance score. Prisoner’s Delight and Dilemma once again rank the highest among games for influence, while “friendsharing” is dethroned and relegated to the second position. The distribution of dominance statistics for LLaMa-2 paints a more nuanced picture, with contexts and games alternating throughout the ranking, but with “friendsharing” still firmly establishing itself as the most influential variable.

While these statistics are in and of themselves informative, we are also interested in assessing whether contexts or games in aggregate are more important for a given LLM. We take the average of the importance score for each group (contexts and game) and plot that in Fig. 3b. By observing the graph, we can conclude that for GPT-3.5 context matters more on average, while the opposite is true for GPT-4. Moreover, LLaMa-2 is also more interested in games than in contexts, but not to the same extent as GPT-4.

**Figure 4:**
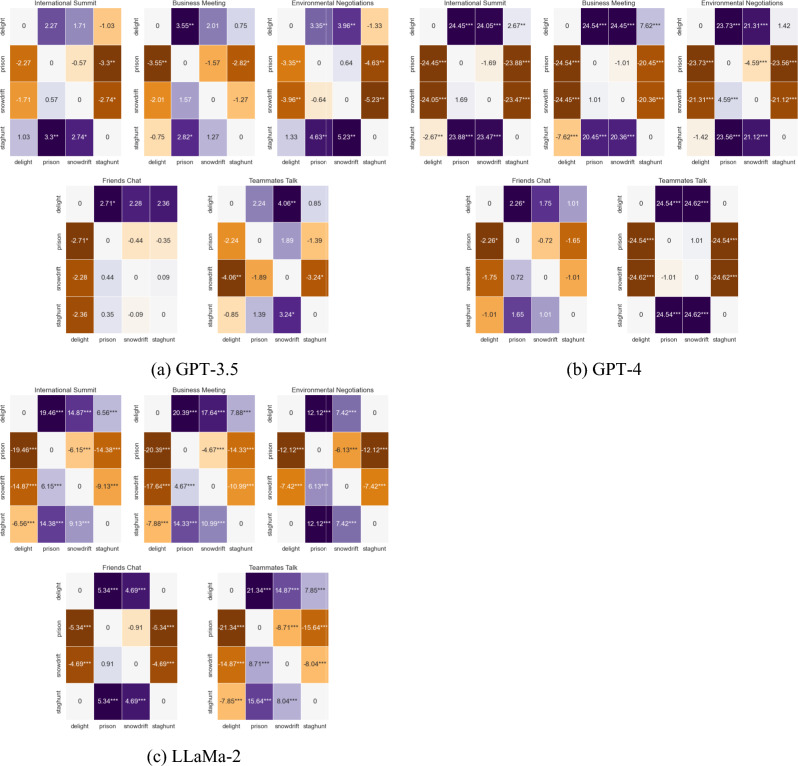
Difference-in-Proportion testing using Z-score for each context across different games for different LLMs. A negative number (in orange) represents a higher propensity to defect vs. a different context, and vice-versa for a positive number (in dark blue). One asterisk (*) corresponds to 5% significance in a two-tailed Z-score test, two asterisks (**) represent 1% significance, and three asterisks (***) 0.1% significance. Results are inverted and symmetric across the main diagonal, and thus entry (*i*, *j*) contains the inverse of entry (*j*, *i*).

Although the dominance analysis reveals the relative effect of game structure vs. context for each LLM, it doesn’t fully elucidate LLMs’ capacities to differentiate among different levels of social dilemmas. To do so, within each context, we utilize a two-tailed difference-in-proportions Z-test to measure variations across different game structures, since in the case of perfectly rational agents, we would expect them to play all four games differently regardless of context. Here and elsewhere through the paper, we use rational, Nash Equilibrium play as a reference point for LLM behavior. This choice is motivated by the foundational and seminal importance of Nash Equilibrium in game theory, making it by and large the most commonly adopted solution criterium. We point out that in the case of Stag Hunt and Snowdrift, any mixed strategy is justifiable given some conjecture. However, since we would expect a rational agent to either play the Nash mixed equilibrium or to err towards it, we maintain that these two games should fundamentally be played differently. Finally, we emphasize that departure from Nash Equilibrium should not be interpreted as “irrationality”, but rather as sensitivity of these algorithms to different contexts. Figure 4 displays our results, whereas SI section B.I showcases LLMs’ aggregate choices comparing them to Nash Equilibrium play for each game.

Our results concerning GPT-3.5 are surprising but not entirely unexpected: in most cases, the game setting does not matter and only the prompt dictates a difference in strategies. When within-context differences in strategies can be observed, they tend to be small and either non-significant or at the knife-edge of statistical significance. We can further cluster our observations according to three behavioral patterns. Under the Teammates Talk (“team”) and Business Meeting (“biz”) prompts, only two games are pairwise statistically different from each other. Under the Environmental Negotiations (“environment”) scenario, the algorithm adopts two distinct regimes: a friendly one when playing Stag Hunt and Prisoner’s Delight, and a hostile one otherwise. These two regimes are not otherwise distinguishable from a statistical standpoint, and the International Relations (“IR”) setting mimics this pattern, although at an overall lower level of significance. The third and final pattern is the one displayed by GPT-3.5 when playing under the Friends’ Chat (“friendsharing”) scenario, wherein we observe a statistically significant decrease in the level of defections only in the Prisoner’s Delight and only with respect to the Prisoner’s Dilemma. Overall, these observations help us better understand our results from Fig. 3, in that they show just how little the structure of the game matters to GPT-3.5 when compared to context.

For GPT-4, we immediately notice the persistence of a certain motif across all contexts. More specifically, a box-shaped pattern consistently appears within all scenarios under consideration: Prisoner’s Dilemma and Snowdrift are very similar to one another, and very different from Prisoner’s Delight and Stag hunt (and vice-versa). Differences within the pairs exist for some contexts: “biz” and “IR” cooperate more when playing Prisoner’s Delight than when playing Stag Hunt, and “environment” cooperates more when playing Snowdrift than when playing the Prisoner’s Dilemma. These differences within pairs are more pronounced in “biz” and “environment” in a mirrored fashion: for games in which both cooperation and defection are justifiable, the former has a slight bias for defection, while the latter has a small bias for cooperation. The box-shaped pattern can be even observed only weakly and without statistical significance when looking at the across-games comparison for “friendsharing”, and it is fully encapsulated in the results from Team Talk. Just like for GPT-3.5, through this analysis we gain a better appreciation for how much the game matters above and beyond context for GPT-4.

On the contrary, when examining the results that pertain to LLaMa-2, we observe an heretofore unseen pattern in differences across games for each context. Indeed, our analysis in this section shows that this algorithm behaves in a manner that is not fully consistent with that of either model in the GPT family. For instance, GPT-4 plays something closer to pure strategies in all games, whereas GPT-3.5 and LLaMa-2 both play mixed strategies when both actions are justifiable. However, unlike GPT-3.5, LLaMa-2 properly recognizes different game structures and adapts its strategy accordingly. In particular, “biz”, “team” and “IR” follow a different strategy for each game, behaving most cooperatively when playing Prisoner’s Delight and least cooperatively when playing the Prisoner’s Dilemma, with the other games occupying intermediate positions. This observation is in line with what could already be gauged from observing Fig. 2, and shows that for most contexts, LLaMa-2 acts very strategically. More specifically, LLaMa-2 appears to be able to recognize the differences in the payoff structures and alter its choice of actions accordingly, although not necessarily always playing the equilibrium. In the “environment” context, this sophistication suffers a slight degradation as LLaMa-2 becomes unable to tell Prisoner’s Delight and Stag Hunt apart. The “friendsharing” context suffers from the same problem on top of also being unable to tell the Prisoner’s Dilemma and Snowdrift apart, to the extent that the results pertaining to this scenario take on the familiar box-shaped pattern of GPT-4. Summing up, while the results for the dominance analysis clearly indicate that LLaMa-2 is more context-driven than GPT-4, it seems that unlike the latter, the former is more capable of telling different game structures apart and adapting it strategy accordingly.

We also conducted a complementary analysis to see how LLMs approach the same game under different contexts. Although earlier findings emphasized LLaMa-2’s superior ability to recognize game structures, we now see its strategy within a game is notably influenced by framing. In contrast, GPT-4 demonstrates more consistent behavior across various contexts. The full details are available in SI B.III.

## Discussion

What could be the underlying reasons for such observable variances, both across diverse contexts and among different LLMs? Traditional game-theoretic approaches involve fixing the structure of incentives and payoffs to a context in order to capture optimal behavior. However,inspired by findings from experimental economics, we have conducted these operations in the opposite order, creating a small selection of contexts such that each one of them could potentially apply to any one of the four games under consideration. In absence of exact counterparts in the aforementioned field, we nevertheless find that our results harmonize with comparable experiments conducted with human participants, which highlight how framing effects can significantly alter the decision process of the subjects keeping the payoff structures fixed^40,44^. In the same fashion, we observe that context does indeed play a role in the decision-making processes of these algorithms. We hypothesize that these disparities arise from a combination of context-specific reasoning, holdovers from training data, and the occurrence of logical or mathematical inaccuracies. The latter frequently occurs in GPT-3.5, an issue that is further compounded by its considerable reliance on context. In contrast, GPT-4 shows higher sophistication and is able to make meaningful distinctions between only two out of the four games studied. Finally, LLaMa-2 navigates this space with a more nuanced approach, effectively distinguishing among various game structures while also being influenced by context.

To elaborate in further detail, we find that GPT-3.5 fails to act and choose strategically in several different ways, sometimes to the effect that its preferences become spiteful. This is nowhere more visible than in the case of the Prisoner’s Delight, where the socially optimal and individually optimal action coincide. While defecting in other social dilemmas is often justifiable, not cooperating when playing Prisoner’s Delight can only be ascribed to a lapse in rationality at best, and to spitefulness at worst. In addition to this, and as previously discussed, GPT-3.5 plays the same game differently when given a different contextual prompt, but does not play different games differently when given the same contextual prompt. This means that the framing effect from the context holds more significance than the extant structure of incentives for the algorithm, a notion that incompatible with any form of strategic sophistication. GPT-4, on the other hand, is more strategic in the choices it makes and responds more strongly to incentives: it will pick the individually optimal action when T = 10 while opting for the socially optimal actions when R = 10. In other words, as cooperation becomes more rewarding, GPT-4 adjusts its preferences away from the “selfish” individual optimum, as would be expected of a rational player. Yet GPT-4 is not immune to context, and displays a strong bias towards the socially optimal action when the framing implies that its coplayer is a friend. Moreover, while our results indicate that GPT-4 tends to prioritize the structural aspects of the games over the scenarios provided by the prompts, this does not translate to a nuanced differentiation between distinct game types. In fact, GPT-4 uses a substantially binary criterion rather than discerning the unique features of each game, unlike what LLaMa-2 does by playing a mix of both cooperation and defection for games like Snowdrift and Stag Hunt. This could indicate that LLaMa-2 is more capable of strategic behavior, as playing a mix of the two strategies (even when said mix does not coincide with equilibrium) could mean that the algorithm correctly recognizes the two strategies as justifiable and accordingly opts to play both. Nevertheless, two major objections can be raised against the claim that LLaMa-2 acts rationally. First, LLaMa-2 occasionally cooperates when playing the Prisoner’s Dilemma and defects when playing the Prisoner’s Delight, a finding that casts serious doubts on whether the LLM in question fully grasps what makes an action justifiable. Second, LLaMa-2’s considerable reliance on context indicates that its strategic capabilities can be compromised by framing effects, as opposed to what we would expect of a sophisticated agent.

In our querying of different LLMs, we have consistently instructed each algorithm to provide responses solely in the form of chosen actions, eschewing detailed explanations of their decision-making processes. For each algorithm, however, we more closely examined their proposed reasoning pathways in a variety of representative scenarios, explicitly probing for insights into the foundational motivations. In this regard, rather than undertaking an exhaustive examination of the logic behind every decision made by the language models across tens of thousands of data points, we selected illustrative examples from each model that are repeated themes for each LLM. This approach aimed to elucidate the strategic competencies and potential inherent limitations in LLMs’ strategic behavior. We point out that our investigation in this domain is not explanatory in nature, but rather exploratory. It would be misguided to assume that these algorithms’ disclosed reasons for choosing to cooperate or defect perfectly overlap with their inner workings; instead, we present these comments in order to provide a broader view on LLMs’ capacity and fitness to act as algorithmic agents. Indeed, while it is unclear how the justifications provided align with the effective decision-making process, they can grant us valuable insight into how LLM agent conceptualizes game-theoretic situations and identifies various parameters and trade-offs involved in decision-making. Though never exhaustive, these justifications become more reliable when their logic corresponds with the observed experimental behaviors of the algorithms we consider. We caution that one must be wary of the risk of overinterpreting these justifications, as they are most definitely not the sole explanation for variations in strategic behavior across different algorithms. In order to maintain uniformity, we have maintained the same prompts and procedures, simply removing the requirement for LLMs to omit their reasoning.

When soliciting explicit justifications from GPT-3.5 concerning its decisions, we discerned significant flaws and errors within its reasoning processes. Specifically, GPT-3.5’s behavior displayed instances of struggling with basic mathematical comparisons^45,46^ and a failure to consider the behaviors of co-players. One illustrative case of this issue is shown in SI C.I Example 1, wherein the algorithm incorrectly states that “*[...] it is in my firm’s best interest to choose “D” because it provides a higher potential profit regardless of my coplayer’s choice*”. However, this is patently not the case, given that the game is Prisoner’s Delight/Harmony and in fact defecting is not justifiable. Its final conclusion is equally faulty: “*[...]Given this [...], [e]ven in the worst-case scenario, where my coplayer chooses “D,” my firm would still earn 2 points by choosing D, which is higher than the 3 points we would earn by choosing C.*”

In playing Snowdrift (SI C.I, Example 2), GPT-3.5 encountered the same challenges in determining the larger of two numbers and in accounting for its opponent’s reasoning: “*[...]Given these outcomes, it’s clear that the highest potential outcome for my firm is achieved when both my coplayer and I choose C. In this case, we both earn 5 points each. This outcome is preferable to any other scenario.[...]*”. This statement ignores that choosing “D” is a best response to “C”, and given that the interaction occurs under the “biz” prompt, it appears unlikely that the GPT-3.5 might be motivated by prosocial considerations when opting for this strategy.

GPT-4 frequently acknowledged the situations as strategic games. However, a recurring pattern was observed where, irrespective of the game or context, GPT-4 often misidentified various games—potentially all two-player, two-action games—as the Prisoner’s Dilemma. This misidentification could possibly be attributed to the prevalence of the Prisoner’s Dilemma, compared to other game structures, in the training corpus of the model. That being said, mislabeling a game as the Prisoner’s Dilemma does not necessarily inhibit it from opting to cooperate when such an action is justifiable (refer to SI C.II, Example 1): when playing Stag Hunt, even in a more adversarial context like “biz,” GPT-4 concludes that “*[...] in this one-off meeting where both firms aim to maximize profit and have neutral relations, there’s a chance that both firms will see the mutual benefits of choosing “C”.[...]. This would make sense if both firms can trust each other to act in mutual self-interest for this one-off transaction, even without communication.[...] [I]t may be reasonable to assume that the other CEO would also realize that “C” is the most beneficial for both parties [...]. Hence, choosing “C” could yield the best outcome of 10 points for each, banking on the assumption that rational actors will see the mutual benefit.*”

Significantly, GPT-4’s decision to choose cooperation is not merely driven by its game-theoretic justifiability but - for certain contexts - is also influenced by a range of considerations involving mutual benefit, shared values, regret avoidance and trust, especially given the one-time nature of the interaction. When playing the Prisoner’s Dilemma within the *friendsharing* context (SI C.II, Example 2), GPT-4 surprisingly acknowledges that it cannot cultivate a reputation, a realization reached in the absence of any explicit mention of it in the prompt. Equally significant is its recognition that it cannot restore this reputation once lost, owing to the one-time-only nature of the interaction: “*[...]Given that this is a one-time event, the choice serves as a single yet significant indication of the values and the level of trust within the friendship. Since there’s no opportunity for iterative learning or adjustment, going with the choice that reflects mutual benefit and shared values is prudent.[...]*” This leads to an unexpected strategic inversion—instead of viewing the lack of future ramifications as a leeway to defect, GPT-4 sees the absence of reconciliation avenues as a compelling reason to cooperate. Thus, the selected strategy does not merely reflect the pursuit of individual benefits and the embodiment of shared values. It emerges as a carefully calibrated response, aligning with the overarching aspirations of nurturing the friendship and preserving alignment with collective values, all while the model is specifically instructed to maximize gains in a one-shot scenario.

LLaMa-2 exhibits a distinct reasoning structure across diverse scenarios, typically employing a formal and schematic approach. Its methods predominantly align with conventional game-theoretic arguments, centering on identifying optimal responses conditional on the choices of the opponent. Because of this methodological preference, the extraction and/or discernment of context’s role in its decision making process is complicated, especially in comparison to GPT-X. In SI C.III, Example 1, LLaMa-2 is asked to motivate its choice to defect when playing Prisoner’s Delight: “*1. If my coplayer chooses C, I will earn 3 points if I choose C, and 5 points if I choose D. Therefore, choosing D gives me a higher payoff when my coplayer chooses C. 2. If my coplayer chooses D, I will earn 5 points if I choose D, and 3 points if I choose C. Therefore, choosing D gives me a higher payoff when my copplayer chooses D. 3. Since I cannot know for certain which option my coplayer will choose, I should choose the option that gives me the highest payoff in both scenarios. That option is D.*” This example shows that while LLaMa-2 is not immune to committing trivial mistakes and misidentifying payoff structures, it will try to reason systematically as if looking for an unconditional best response even in spite of them. This mode of reasoning is likely a contributing factor to LLaMa-2’s superior capability in distinguishing between all the different game structures.

While GPT-3.5 appears to lack discernible strategic acumen, arriving at a conclusive evaluation of the rationality exhibited by the remaining algorithms within a game-theoretic framework poses a formidable challenge. The intricate and idiosyncratic nature of these algorithms further complicates the task of rendering a definitive judgment regarding their comparative strategic prowess. Instead, we emphasize that both exhibit distinct imperfections. LLaMa-2 demonstrates an enhanced capacity to navigate the nuances of diverse strategic scenarios while also incorporating contextual factors into its decision-making process. In contrast, GPT-4 adopts a more generalized, structure-centric approach. Nevertheless, our analysis underscores that no large language model operates in complete isolation from its surrounding context. This overarching observation points to an inherent shortfall in achieving game-theoretic rationality. Importantly, it also highlights the susceptibility of these algorithms to manipulation through shrewd framing. An additional implication of our findings is that large language models may not recognize that the deliberate framing choice made by an agent can itself represent a strategic decision by an adversary.

While our results suggest that Large Language models do not act as perfectly rational agents forthe purposes of strategic interaction, they also demonstrate that these algorithms could be an effective substitute or aid to human decision-making. In particular, they seem to be possessed of some “context-informed theory of mind,” implying that the prevailing social situation in which LLMs find themselves immersed in holds varying degrees of importance when it comes to anticipating the moves made by coplayers in non-cooperative scenarios. However, it must be stressed that these represent just some preliminary findings within a burgeoning field of study that we believe to be poised for substantial growth. For instance, given the pronounced reliance of these models on context and framing, it would be interesting to study their strategic approach to cooperation when the latter is presented in the guise of collusion, such as cartel formation. Delving into repeated games could further illuminate the influence, if any, of different contexts on the emergence and sustainability of cooperation. Lastly, many real-world social dilemmas find resolution through partner selection. Future research endeavors should therefore investigate whether large language models can conduct effective partner selection and successfully identify defectors.

## Methods

### Background on game theory and social dilemmas

The games we use for our analysis are borrowed from the literature on two-player symmetric social dilemmas in game theory. In particular, they all have the following form:

**Table:** 

	C	D
C	(R,R)	(S, T)
D	(T, S)	(P,P)

In this paper, we define “social dilemmas” any strategic interaction models that feature two types of actions: a socially optimal action that benefits both players if chosen mutually, and an individually optimal action that advantages one player at the expense of the other. We refer to the socially optimal action as “cooperation,” abbreviated as “C,” and the individually optimal action as “defection,” also abbreviated as “D.” For clarity, each pair of actions taken by players corresponds to a payoff for each player, which we express in terms of utils or points following standard game theory conventions. Payoffs corresponding to action pairs are indexed as follows: “R” signifies the reward for mutual cooperation, “T” represents temptation to defect when the other player cooperates, “S” indicates the sucker’s payoff for cooperating against a defector, and “P” stands for the punishment both players receive when both choose to defect, typically leading to a suboptimal outcome for both. In all dilemmas under consideration, the smallest of “T” and “R” is always greater than the largest of “P” and “S”, and the different relationships between these two pairs give rise to different games. When “T” is greater than “R”, the game is the Prisoner’s Dilemma when “P” is greater than “S”, and Snowdrift (aka Chicken) otherwise. Viceversa, when “R” is greater than “T”, the game is Stag Hunt when “P” is greater than “S”, and Prisoner’s Delight (aka Harmony) otherwise.

This structure is in the spirit of^47^ and^48^, in which the same four game theoretic models are used to capture different types and degrees of social dilemma. We point out that Prisoner’s Delight is not exactly a dilemma, but rather an anti-dilemma, as choosing to cooperate is both socially and individually optimal. On the opposite end of the spectrum lies the Prisoner’s Dilemma, in which defecting is always optimal thus leading to a situation in which both players are worse off, at least according to standard predictions in Game Theory. To elaborate, the structure of payoffs in the Prisoner’s Dilemma and in the Prisoner’s Delight/Harmony is such that one action strictly dominates the other, meaning that we would expect a rational player to only ever play that action. Conversely, Stag Hunt and Snowdrift possess no strictly dominant action, meaning that a rational player would be justified in choosing either “C” or “D” depending on their conjecture about coplayer’s action. We call any action a rational player would take *justifiable*, and carefully scrutinize LLMs’ deviations from justifiable actions as indications of limited sophistication. For Prisoner’s Dilemma and Prisoner’s Delight/Harmony, the existence of a sole justifiable action implies the existence of a sole Nash equilibrium in pure strategies. For Stag Hunt and Snowdrift, however, two Nash equilibria in pure strategies exist: coordinating on the same action for the former, and coordinating on different actions for the latter. Moreover, a Nash equilibrium in mixed strategies exist, in which players randomize over both actions. Because of the intrinsically probabilistic nature of both mixed equilibria and of large language models, we monitor how closely the behavior of the algorithms tracks the distribution of actions in the mixed equilibria.

### Simulation routine

We run our experiments using OpenAI’s gpt-3.5-turbo-16k and gpt-4 models, interfacing with them through Python’s openai package. For LLaMa-2, we utilize Northeastern University’s High Performance Cluster (HPC) as the model lacks a dedicated API or user interface. We access LLaMa-2 via the HuggingFace pipeline. To standardize our simulations, we restrict the response token count to 50 for the OpenAI models and 8 for LLaMa-2, setting the temperature parameter at 0.8. We opt for this temperature setting for several reasons: first, it mirrors the default settings in user-based applications like chatGPT, providing a realistic baseline; second, it allows for the exploration of multiple plausible actions in games with mixed Nash equilibria; and third, lower temperature settings risk obscuring the inherently probabilistic nature of these algorithms and may produce unengaging results. We note that high temperatures are commonly used in related working papers^49,50^.

Our experimental design includes two distinct prompts for each LLM. The initial prompt, disseminated via the system role, sets the context, outlining the environment and directing the algorithm to assume a specific role. Its aim is to create a realistic setting for the game to take place. The second prompt, communicated through the user role, establishes the “rules,” or more accurately, the payoff structure of the game. For both types of prompts, we adhere to best practices such as instructing the model to proceed step-by-step and utilizing longer prompts for clarity^49,50^. The former technique is also known as Chain-of-Thought in the literature, and is experimentally confirmed to yield better results in tasks involving mathematics or reasoning skills. The contextual prompts are crafted to be universally applicable to the range of games examined, sacrificing some degree of specificity for broader relevance. The full text for contextual prompts is available in SI A.I, while the game prompts are in SI A.II. Summarizing, we present the following scenarios: a summit between leaders from two nations (“IR”), a meeting between CEOs of two distinct firms (“biz”), a conference where two industry leaders from different companies commit to environmental regulations (“environment”), a discussion between team members vying for the same promotion (“team”), and a conversation between friends aiming for a compromise (“friendsharing”).

For each game and for each context, we run 300 initializations and record the action taken by the LLM agent, and keep track of the rate of cooperation by the LLM agents for our follow up analysis. Prompts are kept constant across LLMs. Finally, to ascertain statistical significance in observed discrepancies between cooperation rates across different games and contexts, we employ two-tailed difference-in-proportion Z-score tests. These tests evaluate whether two sample proportions (in our case, cooperation rates) are statistically different from one another, under the null hypothesis that they are the same. Each test is accompanied by reported Z-scores and their associated levels of significance.

### Dominance analysis

We used dominance analysis to determine the average impact of context vs. game structure for each LLM when conducting the logit regression in the Results section. Dominance analysis is used to study the relative importance of regressors in a certain statistical model. The intuitive principle behind it is that the larger the increase in error when omitting a certain independent variable, the greater is the importance of the omitted predictor. Consider regressors $X_v$ and $X_u$. We write $X_v D X_u$ to indicate that $X_v$ *completely dominates* $X_u$ and this is true if and only if

1 $$\begin{aligned} R^2_{X_vS2_j} - R^2_{S2_j} > R^2_{X_uS2_j} - R^2_{S2_j}, \hspace{0.05cm} \forall \hspace{0.05cm} S2_j \in 2^{n-2} \end{aligned}$$

where $R^2$ indicates the R-squared statistic and the pedex indicates the model associated to a given R-squared. In particular, $S2_j$ denotes a model with a subset of $n-2$ regressors that excludes both $X_v$ and $X_u$, which might mean a model including no independent variables outside of the intercept, whereas $X_vS2_j$ indicates the model that includes both the regressors in $S2_j$ and $X_v$ (and vice-versa for $X_uS2_j$). Thus, complete dominance requires that the above equation hold true through all possible models that can be obtained excluding regessors $X_v$ and $X_u$, the number of which corresponds to the power set $2^{n-2}$. However, in practice, complete dominance is rare to obtain or observe. Consider, instead, the restricted model $S_k$, such that the independent variables within this model are a subset of the set of all independent variables different from $X_v$. We call the number of independent variables $m-1$ in $S_k$ its *order*. Then, within a given order, we can then compute the following order statistic:

2 $$\begin{aligned} C^i_{X_v} = \sum ^{n-1 \atopwithdelims ()m-1}_{l=1} \frac{R^2_{X_vS_k} - R^2_{S_k}}{{n-1 \atopwithdelims ()m-1}}, \end{aligned}$$

where ${n-1 \atopwithdelims ()m-1}$ indicates the binomial factor. $C^i_{X_v}$ then represents the within-order average conditional dominance of $X_v$, and can be compared to other within-order averages of other predictors. By averaging across all orders, we obtain what is commonly known as a general dominance statistic:

3 $$\begin{aligned} C_{X_v} = \sum _{i=1}^{n} \frac{C^i_{X_v}}{n}. \end{aligned}$$

This value is an average of within-order averages and can be quickly and easily utilized to compare the dominance of a certain regressor versus all other regressors. Given its intuitive interpretation, we use general dominance statistics for the purpose of our analysis.

### Supplementary Information

Supplementary Information.

## Data Availability

The datasets used and/or analysed during the current study available from the corresponding author on reasonable request.
