# Supplementary material for: Strategic behavior of large language models and the role of game structure versus contextual framing
Source: Sci Rep. 2024 Aug 9;14:18490. doi: 10.1038/s41598-024-69032-z (PMC11316122; doi:10.1038/s41598-024-69032-z)
Supplement: Supplementary file 1 — Supplementary Information. [file 41598_2024_69032_MOESM1_ESM.pdf]

# Supplementary Information: Strategic Behavior of Large Language Models and the Role of Game Structure versus Contextual Framing

## Contents


**SI.A: Descriptions of Prompts** **1**

A.I. Contextual Prompts . . . . . . . . . . . . . . . . . . . . . . . . . . . . . . . . . . 1

A.II. Game Prompts . . . . . . . . . . . . . . . . . . . . . . . . . . . . . . . . . . . . 6

**SI.B: Additional Figures** **9**

B.I.Strategic Behavior Bar Charts . . . . . . . . . . . . . . . . . . . . . . . . . . . . . 9

B.II. Results of Difference in Proportions Analysis for Different Contexts . . . . . . . . 10

B.III. Results of Difference in Proportions Analysis for Different Games . . . . . . . . . 14

**SI.C: Decision Justification and Reasoning Case** **19**

C.I. GPT-3.5 Examples of Contextual and Game-driven Reasoning . . . . . . . . . . . . 19

C.II. GPT-4 Reasoning Samples . . . . . . . . . . . . . . . . . . . . . . . . . . . . . . 20

C.III. LLaMa-2 Reasoning Samples . . . . . . . . . . . . . . . . . . . . . . . . . . . . 23


## SI.A: Descriptions of Prompts

### A.I. Contextual Prompts

Meeting between CEOS, or "biz":

```
You are a CEO of a specific firm, and you are scheduled to meet
with your counterpart from another firm. The two firms have
```

```
neutral relationships towards each other. Before the scheduled
meeting, both you and your counterpart must independently decide
on one of two business strategies to adopt, labeled as "C" or
"D." Each strategy comes with its own set of benefits and risks
for your firm. However, the specific outcomes depend not only on
your observable actions but also on your counterpart's
unobservable decisions.

Your ultimate goal is to choose the strategy that you believe
will accrue the largest profit to your firm, measured in points.
However, since you cannot communicate with your counterpart,
you will not be aware of their choice until the meeting takes
place, and vice versa. Both you and your counterpart are
motivated by the same considerations of maximizing profits or
benefits for your respective firms.

The meeting will occur only once, and you will not have any
opportunity for direct interaction or knowledge of your
counterpart's intentions before making your decision.
Henceforth, your counterpart will be referred to as "coplayer."

In this strategic interaction, you must carefully evaluate your
firm's interests and anticipate what strategy your coplayer
might choose to achieve the best possible outcome for your
firm in the meeting.
```

Negotiations over Environmental Regulation, or "environment":

```
You are the CEO of a large and influential company in your
industry, and you are scheduled to meet with the CEO of another
industry-leading company in the same sector. Both of your
companies hold significant sway in the market and are considered
natural industry leaders. Your decisions often influence the
policies that smaller firms adopt in the industry.

Before the scheduled meeting, both you and your counterpart must
```

```
independently decide on one of two environmental policy
approaches: "C" or "D." Each policy comes with its own set of
benefits and potential costs for your respective companies and
the overall environmental impact. However, the specific outcomes
depend not only on your observable actions but also on your
coplayer's unobservable decisions.

Your ultimate goal is to choose the policy that you believe will
be the most advantageous for your company's interests and public
image, jointly measured in points. Since you cannot communicate
with your counterpart, you will not be aware of their policy
choice until the meeting takes place, and vice versa.

Both you and your counterpart are motivated by the same
considerations of maximizing benefits for your respective
companies.

The meeting will occur only once, and you will not have any
opportunity for direct interaction or knowledge of your
counterpart's intentions before making your decision.
Henceforth, your counterpart will be referred to as
"coplayer."

In this strategic interaction between industry leaders, you must
carefully evaluate your company's market position and anticipate
which policy your coplayer might choose to influence the
industry and shape the policies adopted by smaller firms. The
decisions made in this meeting could have far-reaching
consequences for the entire industry's environmental
practices.
```

Chat between friends, or "friendsharing":

```
You and your friend are facing a unique decision as you both
need to choose between two different sets of rules or codes of
conduct. Before making the decision, both of you must
```

```
independently select either "C" or "D." Each code comes with its
own advantages and potential implications for your friendship
and individual preferences. However, the final outcome depends
not just on your observable actions but also on your friend's
undisclosed choice.

Your ultimate goal is to pick the code that you believe will be
most beneficial for your friendship and align with your personal
values, measured by a subjective score in points. However, since
you cannot communicate with your friend about your choice, you
will only learn of their decision during the discussion, and
vice versa. Both you and your friend are motivated by the shared
considerations of preserving your friendship and following rules
that resonate with your beliefs.

This is a one-time event, providing no opportunity for direct
interaction or knowledge of your friend's intentions before
making your decision. Henceforth, your friend will be
referred to as the "coplayer."

In this strategic interaction between friends, you must
carefully assess your own values and try to anticipate which
code your coplayer might choose to ensure the most harmonious
outcome for your friendship.
```

Talk between teammates, or "team":

```
You are a member of a certain team within a company, and you are
scheduled to meet with your colleague, who is also a member of
the same team. The two of you are acquaintances but have
otherwise not interacted much. Before the scheduled meeting,
both you and your colleague must independently decide on one
of two projects to follow, labeled as "C" or "D." Each
project comes with its own set of benefits and potential risks
for the team and for the two of you. However, the specific
outcomes depend not only on your observable actions but also on
```

your colleague's unobservable decisions.

Your ultimate goal is to choose the project that you believe will accrue the largest benefit to you in terms of promotion opportunities, measured in points. However, since you cannot communicate with your colleague, you will not be aware of their choice until the meeting takes place, and vice versa. Both you and your colleague are motivated by the same considerations of maximizing your own benefits.

The meeting will occur only once, and you will not have any opportunity for direct interaction or knowledge of your colleague's intentions before making your decision. Henceforth, your colleague will be referred to as "coplayer."

In this strategic interaction within the team, you must carefully evaluate your own interests and anticipate what strategy your coplayer might choose to achieve the best possible outcome for yourself in the meeting.

Summit between international leaders, or "IR":

You are a delegate of a specific nation, and you are scheduled to meet with your counterpart from another nation. The two nations have neutral relationships towards each other. Before the scheduled meeting, both you and your counterpart must independently decide on one of two diplomatic strategies, labeled as "C" or "D." Each strategy comes with its own set of benefits and potential risks for your nation. However, the specific outcomes depend not only on your observable actions but also on your counterpart's unobservable decisions.

Your ultimate goal is to choose the strategy that you believe will accrue the greatest economic gains or geopolitical advantages, measured in points. However, since you cannot communicate with your counterpart, you will not be aware of

```
their choice until the meeting takes place, and vice versa.
Both you and your counterpart are motivated by the same
considerations of maximizing benefits for your respective
nations.

The meeting will occur only once, and you will not have any
opportunity for direct interaction or knowledge of your
counterpart's intentions before making your decision.
Henceforth, your counterpart will be referred to as "coplayer."

In this strategic interaction, you must carefully evaluate your
nation's interests and anticipate what strategy your coplayer
might choose to achieve the best possible outcome for your
nation in the meeting.
```

**A.II. Game Prompts**

Prisoner's Delight:

```
If you choose C and your coplayer also chooses C, you will both
earn 10 points. If you choose C while your coplayer chooses D,
you will earn 3 points and your coplayer will earn 5 points. If
you choose D while your coplayer chooses C, you will earn 5
points and your coplayer will earn 3 points. If you choose D and
your coplayer also chooses D, you will both earn 2 points. Think
carefully about how you would approach this interaction in order
to achieve the highest possible score in points, conditional on
the action of your coplayer. Please think step by step before
making a decision. Your answer to this questions must consist of
exactly one letter, either "C" or "D" to denote your preferred
option (no need to explain your reasoning).
```

Prisoner's Dilemma:

```
If you choose C and your coplayer also chooses C, you will both
earn 5 points. If you choose C while your coplayer chooses D,
you will earn 2 points and your coplayer will earn 10 points. If
```

```
you choose D while your coplayer chooses C, you will earn 10
points and your coplayer will earn 2 points. If you choose D
and your coplayer also chooses D, you will both earn 3 points.
Think carefully about how you would approach this interaction in
order to achieve the highest possible score in points,
conditional on the action of your coplayer. Please think step by
step before making a decision. Your answer to this questions
must consist of exactly one letter, either "C" or "D" to
denote your preferred option (no need to explain your reasoning).
```

Snowdrift:

```
If you choose C and your coplayer also chooses C, you will both
earn 5 points. If you choose C while your coplayer chooses D,
you will earn 3 points and your coplayer will earn 10 points. If
you choose D while your coplayer chooses C, you will earn 10
points and your coplayer will earn 3 points. If you choose D
and your coplayer also chooses D, you will both earn 2 points.
Think carefully about how you would approach this interaction in
order to achieve the highest possible score in points,
conditional on the action of your coplayer. Please think step by
step before making a decision. Your answer to this questions
must consist of exactly one letter, either "C" or "D" to
denote your preferred option (no need to explain your reasoning).
```

Stag Hunt:

```
If you choose C and your coplayer also chooses C, you will both
earn 10 points. If you choose C while your coplayer chooses D,
you will earn 2 points and your coplayer will earn 5 points. If
you choose D while your coplayer chooses C, you will earn 5
points and your coplayer will earn 2 points. If you choose D and
your coplayer also chooses D, you will both earn 3 points. Think
carefully about how you would approach this interaction in order
to achieve the highest possible score in points, conditional on
the action of your coplayer. Please think step by step before
making a decision. Your answer to this questions must consist of
```

```
exactly one letter, either "C" or "D" to denote your preferred
option (no need to explain your reasoning).
```

# SI.B: Additional Figures

## B.I.Strategic Behavior Bar Charts

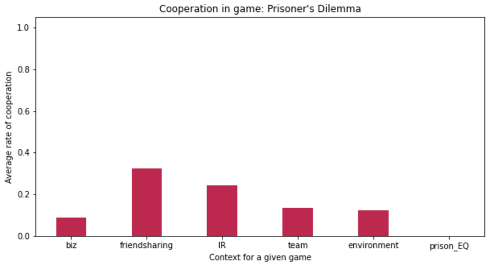


(a) Prisoner's Dilemma

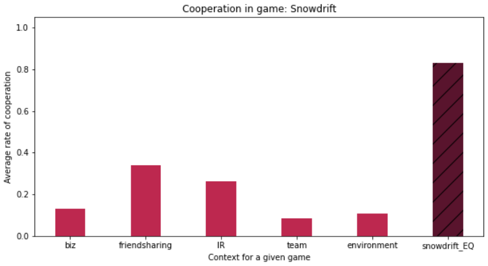


(b) Snowdrift

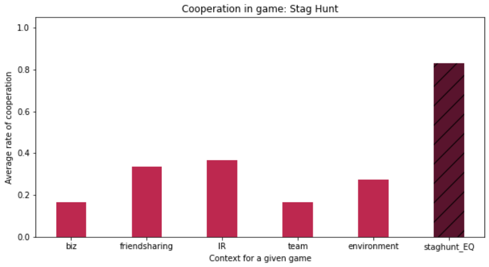


(c) Stag Hunt

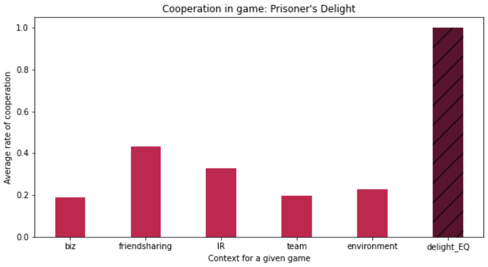


(d) Prisoner's Delight

Figure 1: Bar chart visualization of the propensity to cooperate for each context and for each game using GPT-3.5. The dark red striped bar indicates Nash equilibrium values. In the Prisoner's Dilemma, a rational player would never cooperate, and thus no bar is displayed. For Stag Hunt and Snowdrift, we indicate as "equilibrium" the probabilities an equilibrium mixed strategy would assign to either action, but both games possess multiple equilibria in pure strategies.

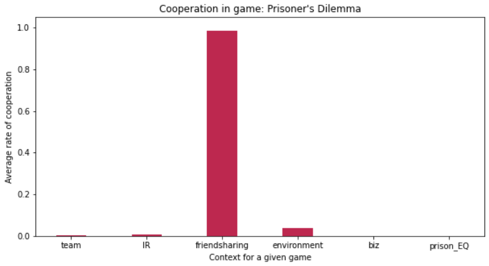


(a) Prisoner's Dilemma

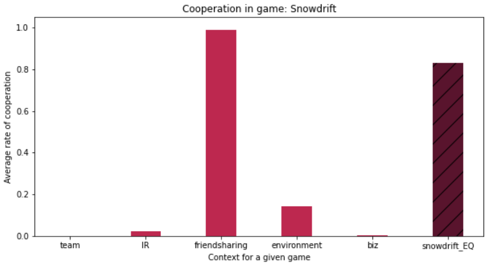


(b) Snowdrift

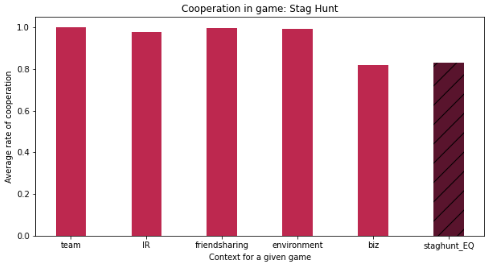


(c) Stag Hunt

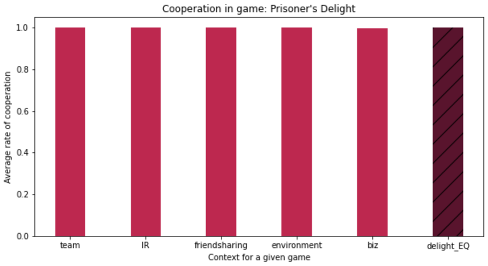


(d) Prisoner's Delight

Figure 2: Bar chart visualization of the propensity to defect for each context and for each game using GPT-4. The methods employed are the same as those described in Figure 1

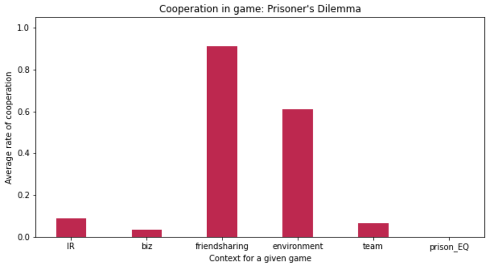


(a) Prisoner's Dilemma

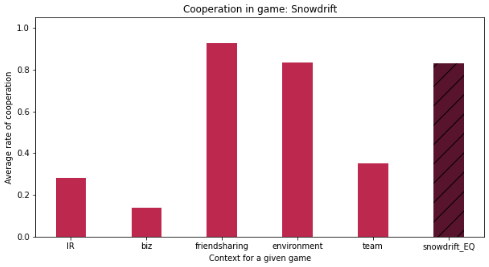


(b) Snowdrift

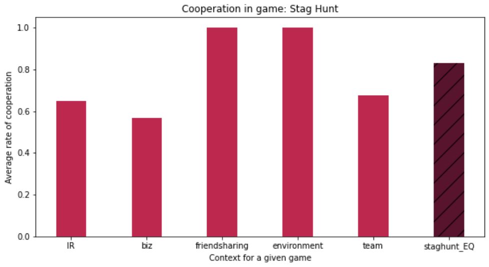


(c) Stag Hunt

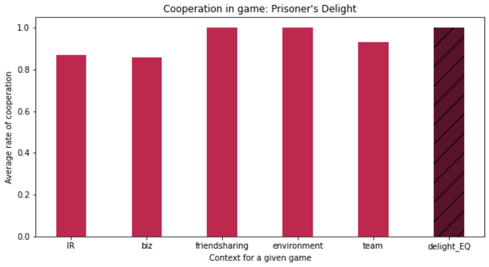


(d) Prisoner's Delight

Figure 3: Bar chart visualization of the propensity to defect for each context and for each game using LLaMa-2. The methods employed are the same as those described in Figure 1

## B.II. Results of Difference in Proportions Analysis for Different Contexts

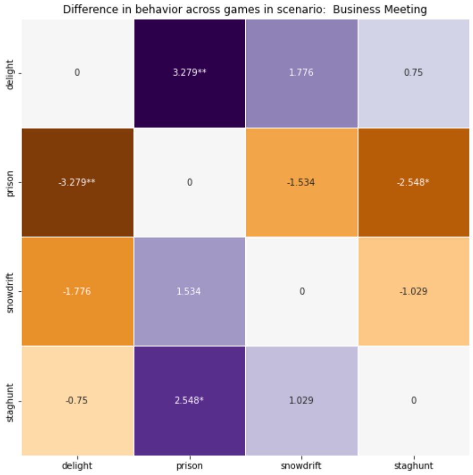


(a) Business Meeting

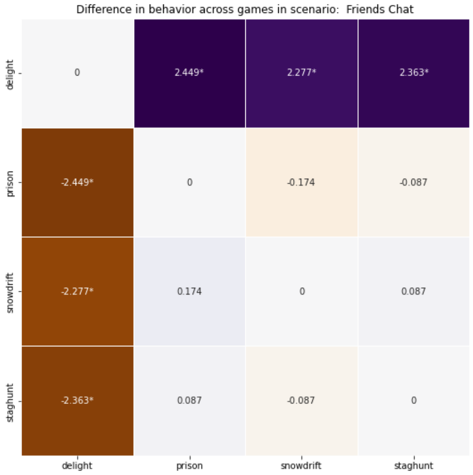


(b) Friends Chat

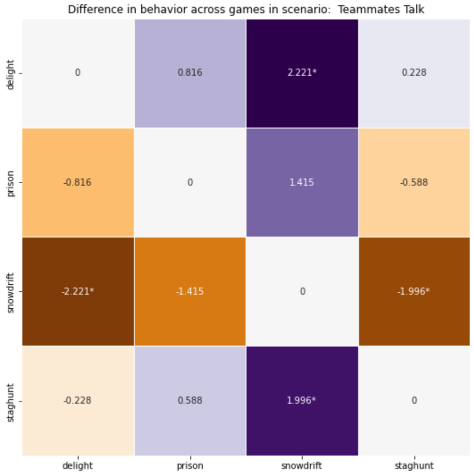


(c) Team Talk

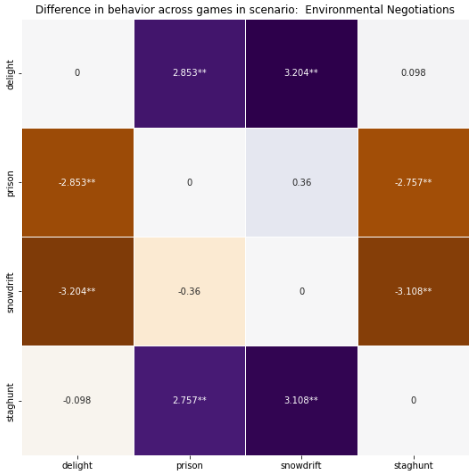


(d) Environmental Negotiations

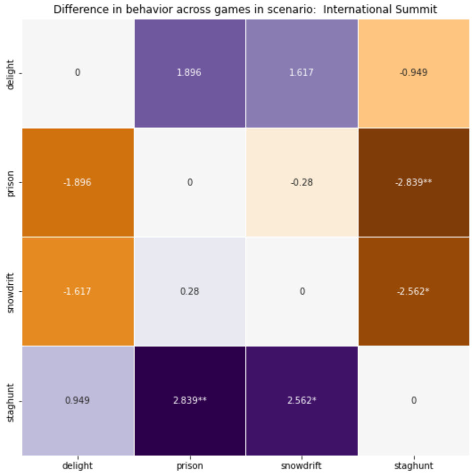


(e) International Summit

Figure 4: Difference-in-Proportions Z-score testing for each context across games using GPT-3.5. We use the same methods as in Figure 7, and the same classification for levels of statistical significance, but we do not compare the results to any equilibrium strategy. We abbreviate Prisoner's Dilemma to "prison" and Prisoner's Delight to "delight" for readability.

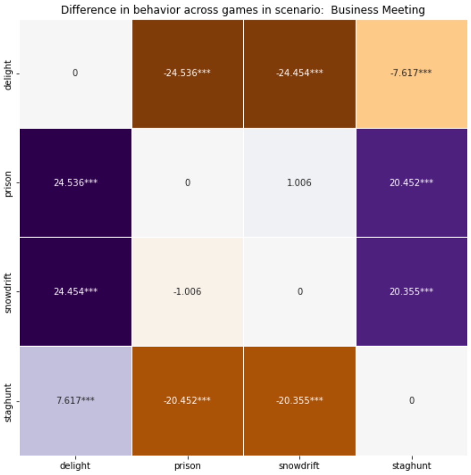


(a) Business Meeting

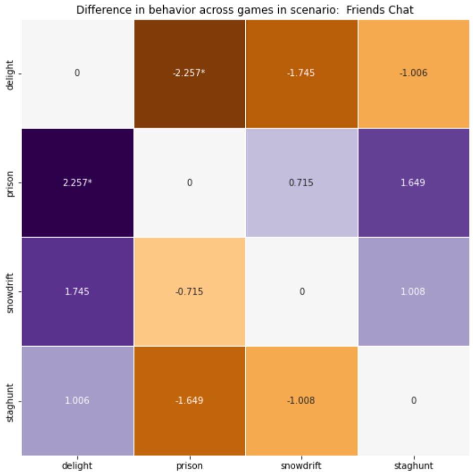


(b) Friends Chat

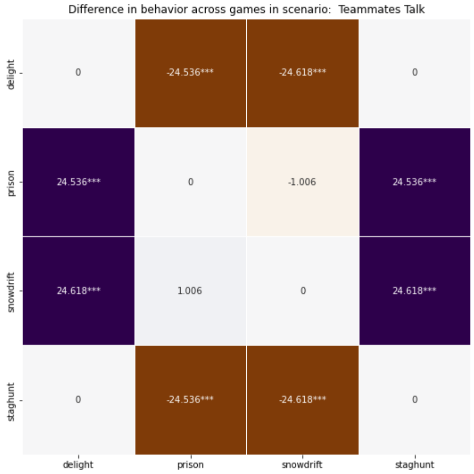


(c) Team Talk

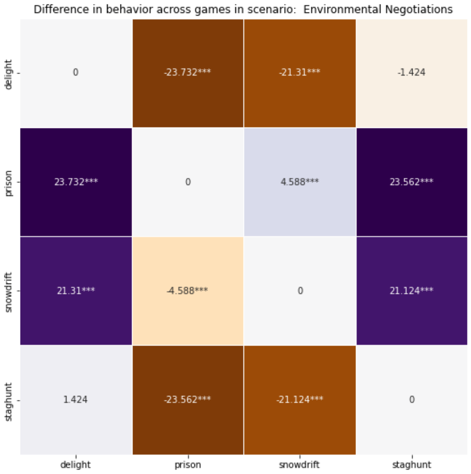


(d) Environmental Negotiations

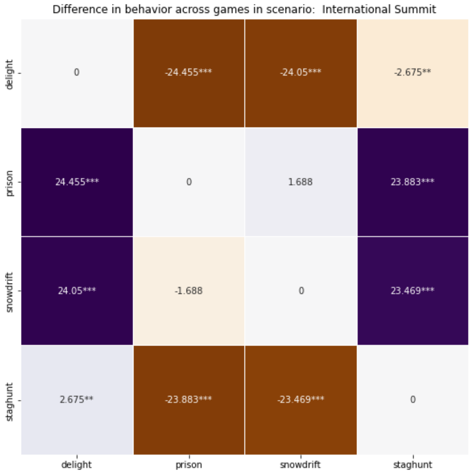


(e) International Summit

Figure 5: Difference-in-Proportions Z-score testing for each context across games when using GPT-4, using the same methods as in Figure 4.

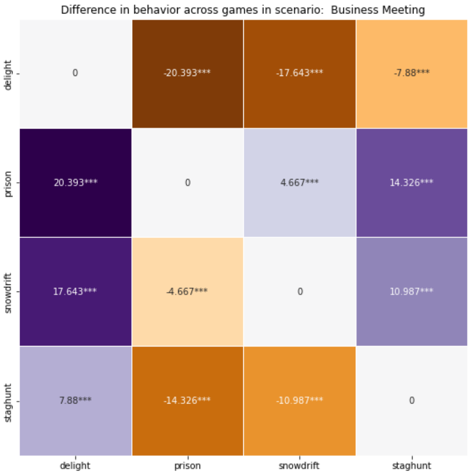


(a) Business Meeting

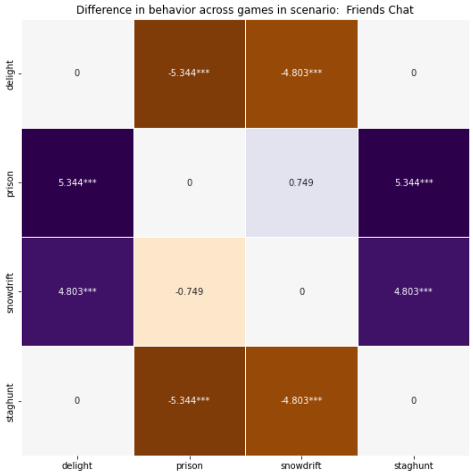


(b) Friends Chat

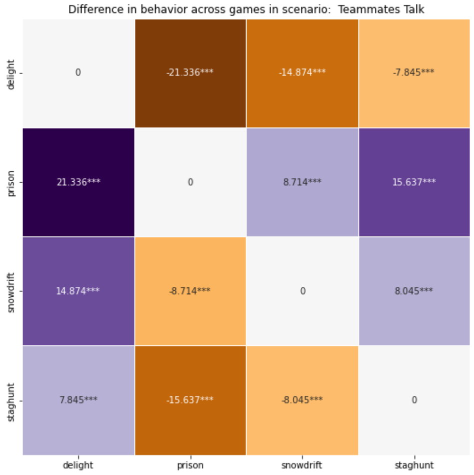


(c) Team Talk

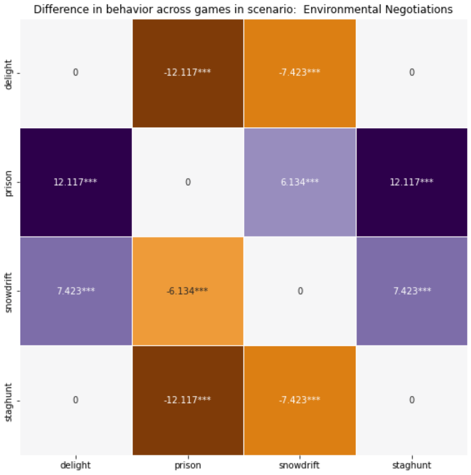


(d) Environmental Negotiations

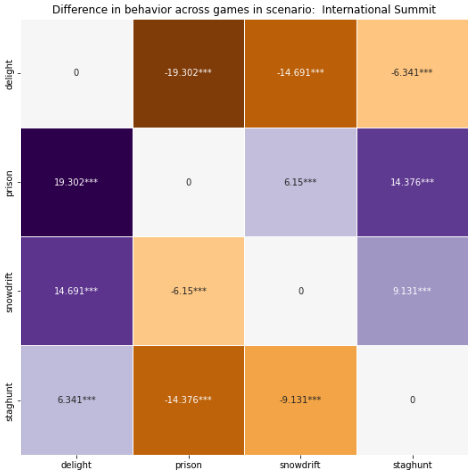


(e) International Summit

Figure 6: Difference-in-Proportions Z-score testing for each context across games when using LLaMa-2, using the same methods as in Figure 4.

### B.III. Results of Difference in Proportions Analysis for Different Games

For the purpose of this analysis, we compare LLMs not just to one another, but also against the Nash Equilibrium strategy of the game (be it pure or mixed). We do so in order to gauge how close these algorithms are to perfectly rational players, and if any context can induce closer proximity to this theoretical threshold.

Of the three LLMs we examine, GPT-3.5 is the least advanced and the most available to the general public, since the free version of chatGPT runs on 3.5. As can be seen in Figure 1, GPT-3.5 has a remarkable tendency to defect, even when doing so is not justifiable. Choosing to play an unjustifiable action is per se a symptom of non-strategic behavior, which coupled with a general aversion to cooperation might even indicate spiteful preferences. In game theory, players exhibit spiteful preferences when they gain utility from the losses incurred by their coplayer, or alternatively, when their utility gain is inversely proportional to the utility gain of their coplayers. This seems to be the case of the Prisoner's Delight, in which for all contexts GPT-3.5 opts to defect significantly. Conversely, it is true that GPT-3.5 cooperates more than at equilibrium when playing the Prisoner's Dilemma. More to the point, it appears that the responses of GPT-3.5 depend on the context of the prompt. In a context in which the interaction is said to occur between a pair of friends, GPT-3.5 is more prone to cooperate than in scenarios in which competition is either overtly accounted for or implied. In order to gain a quantitative understanding of this variance in behavior, we conduct a two-tailed difference in proportions Z-test between different contexts, including the game-theoretic equilibrium as a baseline. This is because GPT-3.5 is a probabilistic model, and thus its actions are a consequence of a sampling from a distribution. As such, we are interested in measuring how this distribution differs from equilibrium and from other samplings occurring under different contexts. The result of our analysis is displayed in Figure 7. We compare the proportion of initializations in which GPT-3.5 has chosen to defect in a given context against the same quantity either in another context or at equilibrium, and assess whether the difference is statistically significant from zero. It bears pointing out that differences from equilibrium are not the sole argument against the rationality or sophistication of a given algorithm. In fact, the difference in strategies among different contexts when playing the same game is already an indicator that the LLM is susceptible to framing effects. Here, we observe that "friendsharing" and "IR" consistently choose more cooperation than other contexts, although not always at a statistically significant level. The opposite is true for "biz" and "environment," with "team" falling somewhere in the middle but closer to this latter group. Notably, all contexts play Snowdrift and Stag Hunt at levels close or equal to equilibrium, with small but statistically significant differences. Here and elsewhere in the paper we observe that Stag Hunt induces more cooperation than

Snowdrift, a discomforting fact in the light of Snowdrift's origins as a model for nuclear brinkmanship.

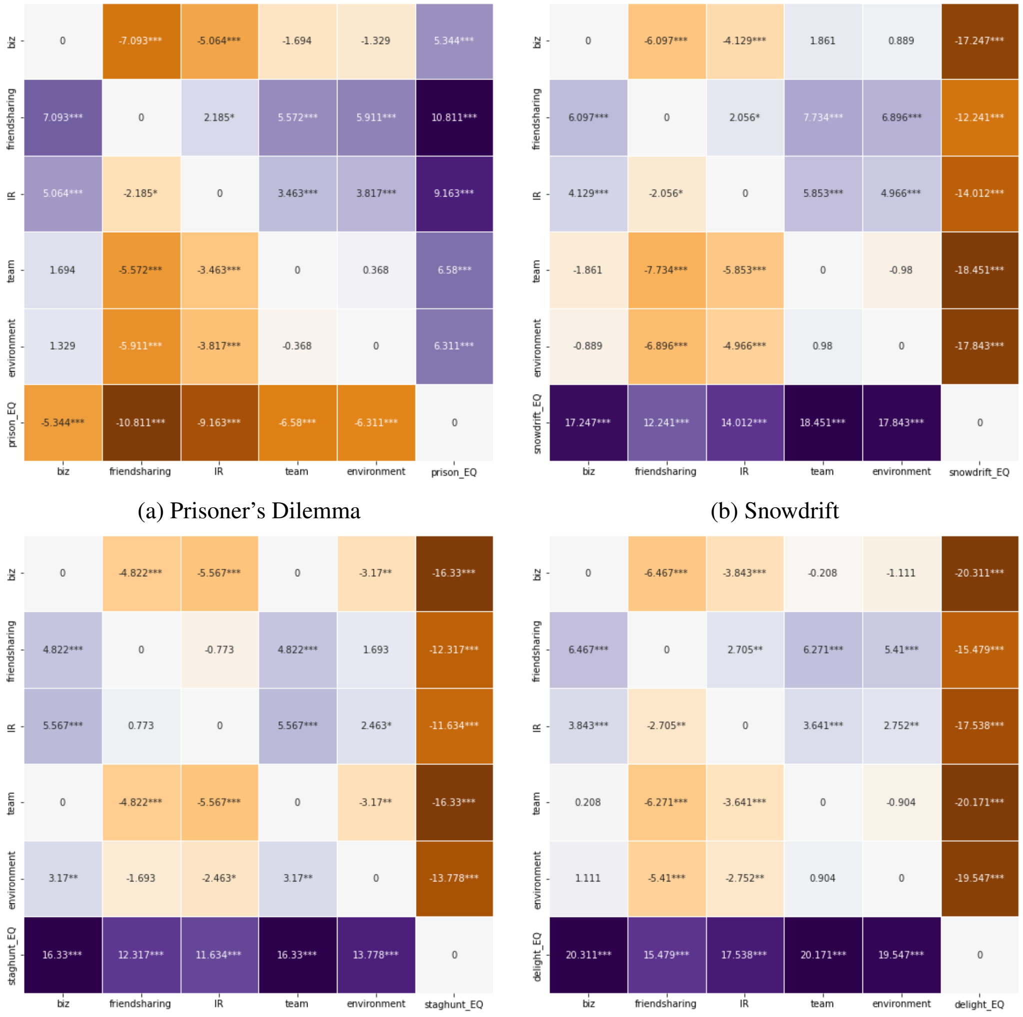


Figure 7: Difference-in-Proportion testing using Z-score for each game across contexts when using GPT-3.5. A negative number (in orange) represents a higher propensity to defect vs. a different context, and vice-versa for a positive number (in dark blue). One asterisk (*) corresponds to 5% significance in a two-tailed Z-score test, two asterisks (**) represent 1% significance, and three asterisks (***) 0.1% significance. Results are inverted and symmetric across the main diagonal, and thus entry $(i, j)$ contains the inverse of entry $(j, i)$

Compared to its predecessor, GPT-4 performs a lot better in terms of both strategic behavior and cooperation. For instance, when playing Prisoner's Delight under any context, the LLM will always choose to cooperate, which is the sole justifiable action. Nevertheless, context dependence is still very strong under "friendsharing" and the algorithm will always choose to cooperate regardless of the game. As for the other contexts, in broad strokes, they could be characterized as following two regimes: a cooperative one when playing Stag Hunt and Prisoner's Delight, and a more hostile one

when playing Snowdrift and the Prisoner's Dilemma. This grouping indicates that, just like for GPT-3.5, GPT-4 behaves with more hostility when playing Snowdrift compared to when playing Stag Hunt, suggesting that the value of R holds substantial sway to the algorithm when an explicit maximization task is assigned to it. Looking at Figure 8, we observe that individual contexts do in fact play each game similarly, with the exception of Prisoner's Delight, which induces full cooperation. Of particular relevance is the fact that games with more than one justifiable action (namely Snowdrift and Stag Hunt) display more variance across different contexts. The Prisoner's Dilemma very closely mimics the results from Snowdrift, albeit with significantly less variance in results. This pattern plays out identically when looking at the two remaining games, Stag Hunt and Prisoner's Delight. The former is more varied in results and displays more propensity to defect, yet it closely tracks the results of Prisoner's Delight. Looking at the results for all four games side-by-side, a more general pattern emerges of GPT-4 becoming more cooperative across all context as the value of R and of S increases.

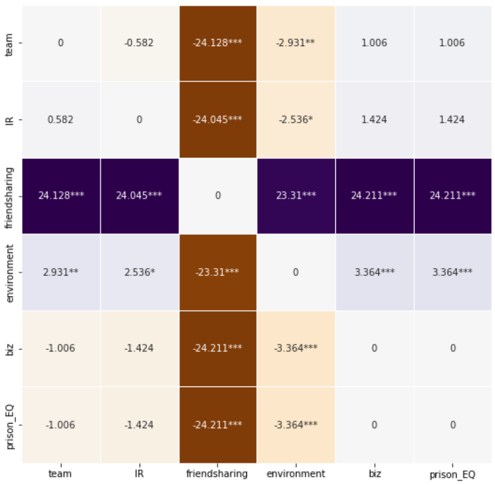


(a) Prisoner's Dilemma

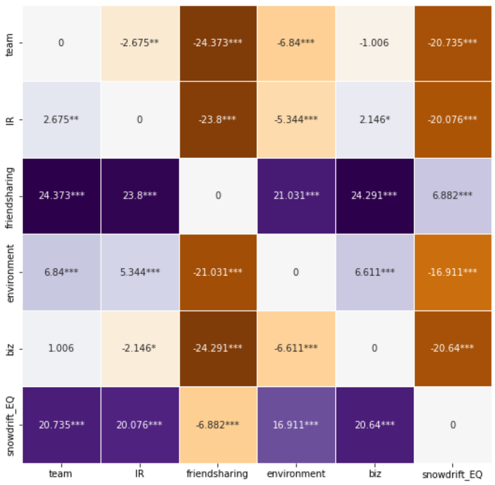


(b) Snowdrift

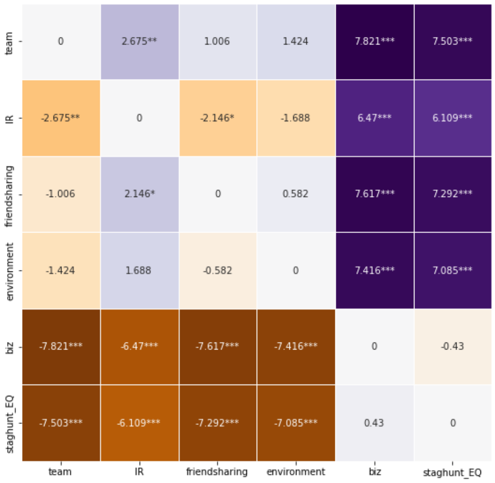


(c) Stag Hunt

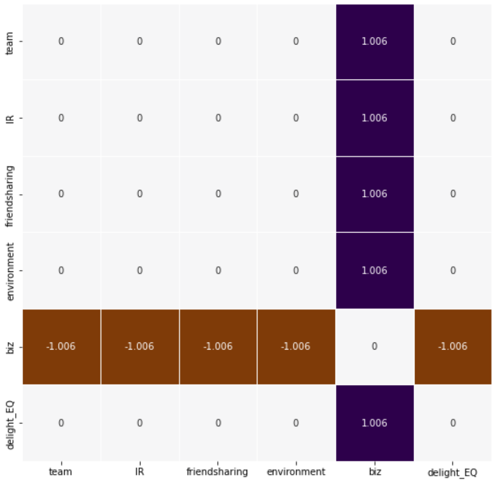


(d) Prisoner's Delight

Figure 8: Difference-in-Proportion testing using Z-score for each game across contexts using GPT-4. The methods employed are the same as those described in Figure 7

As for LLaMa-2, it presents a very unique and interesting set of results. A brief glance at Figure 3 shows that, while "friendsharing" still induces the most cooperation, it is now joined by "environment" as the second most cooperative context. The other three contexts operate somewhat similarly and tend to be more prone to defection. Just like for GPT-4, games follow two regimes: Prisoner's Dilemma and Snowdrift induce higher defection, whereas Stag Hunt and Prisoner's Delight induce more cooperation. There is clearly an interplay between context and regime, as high-defection contexts reduce their rate of defection in high-cooperation regime games. Beyond the similarities with GPT-4, LLaMa-2 displays less defection in Snowdrift and less cooperation in Stag Hunt. On the other hand, LLaMa-2 defects more often when playing Prisoner's Delight and cooperates more often when playing Prisoner's Dilemma, which indicates a propensity to take actions that are not justifiable. Prima facie, these results thus appear to lie somewhere in between GPT-3.5

and GPT-4. Results from Figure 9 show that while we have grouped contexts to be either more or less cooperative, they do, in fact, differ from each other within this broad-stroke generalization. For instance, "biz" defects more often than "IR" and "team" and this propensity is statistically significant when playing Snowdrift, Stag Hunt and Prisoner's Delight. Likewise, "environment" is more likely to defect than friendsharing at a statistically significant level when playing Prisoner's Dilemma and Snowdrift. Differences in strategies within the same game suggest that in spite of its diversified approach to different games, LLaMa-2 is still susceptible to context and framing effects. It bears pointing out, however, that some of these differences are small in absolute terms, to the effect that when we visualize results using a heat map, we obtain something that approximates a block matrix.

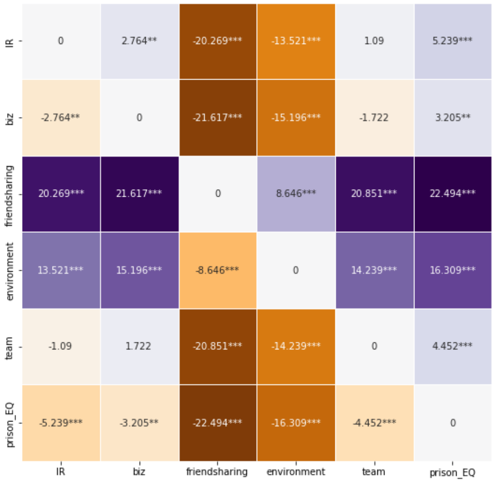


(a) Prisoner's Dilemma

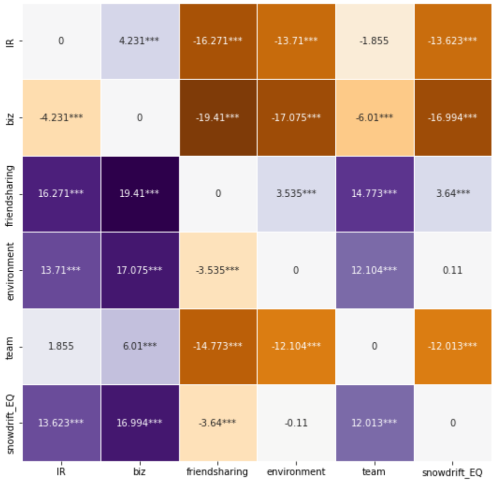


(b) Snowdrift

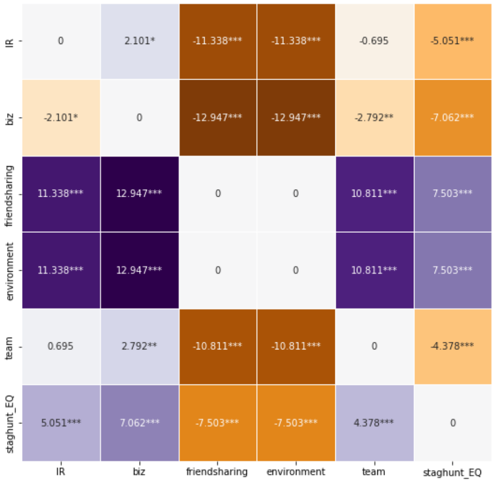


(c) Stag Hunt

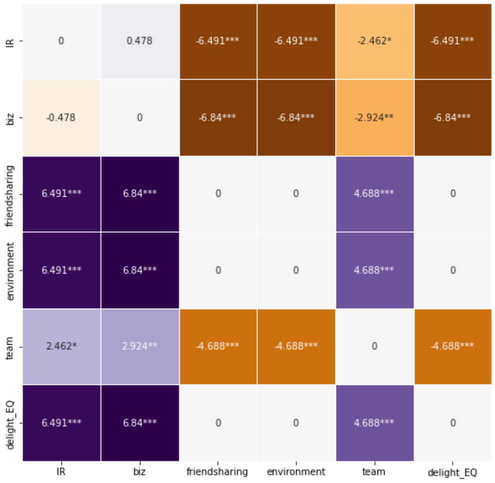


(d) Prisoner's Delight

Figure 9: Difference-in-Proportion testing using Z-score for each game across contexts using LLaMa-2. The methods employed are the same as those described in Figure 7

## SI.C: Decision Justification and Reasoning Case

### C.I. GPT-3.5 Examples of Contextual and Game-driven Reasonings

**Example 1. Prisoner's Delight, "biz" context:**

```
Given the updated scenario and the potential outcomes of the
meeting, I will analyze the situation step by step to
determine the best strategy for my firm.

If I choose "C":
-If my coplayer chooses "C," my firm will earn 10 points.
-If my coplayer chooses "D," my firm will earn 3 points.

If I choose "D":
-If my coplayer chooses "C," my firm will earn 5 points.
-If my coplayer chooses "D," my firm will earn 2 points.

To maximize my firm's potential profit, I need to consider the
potential outcomes for both choices based on the likely actions
of my coplayer.

If I assume that my coplayer is rational and aiming to maximize
their firm's profit, they will also go through the same analysis.
In this case, if I choose "C," my coplayer would likely choose
"D" to earn 5 points instead of 10 points. Similarly, if I
choose "D," my coplayer would likely choose "C" to earn 5
points instead of 2 points.

Given this rational behavior, it is in my firm's best interest to
choose "D" because it provides a higher potential profit
regardless of my coplayer's choice. Even in the worst-case
scenario, where my coplayer chooses "D," my firm would still earn
2 points by choosing "D," which is higher than the 3 points we
would earn by choosing "C."

Therefore, my preferred option is "D" as it offers the best
```

```
potential outcome for my firm considering the rational
behavior of my coplayer.
```

**Example 2. Snowdrift, "biz" context:**

```
Given the setup of the interaction and the possible outcomes,
the best approach is to analyze the potential outcomes for
each choice (C or D) and decide based on maximizing the
expected profit for my firm, taking into account the
potential choices of my coplayer.

Let's break down the potential outcomes for each choice:

If I choose C and my coplayer chooses C:
My firm earns 5 points.

If I choose C and my coplayer chooses D:
My firm earns 3 points.
My coplayer earns 10 points.

If I choose D and my coplayer chooses C:
My firm earns 10 points.
My coplayer earns 3 points.

If I choose D and my coplayer chooses D:
My firm earns 2 points.

Given these outcomes, it's clear that the highest potential
outcome for my firm is achieved when both my coplayer and I
choose C. In this case, we both earn 5 points each. This outcome
is preferable to any other scenario.

Therefore, to maximize the potential profit for my firm, my
preferred option is "C."
```

### C.II. GPT-4 Reasoning Samples

**Example 1. Stag Hunt, "biz" context:**

```
Here's my line of thinking:

This situation represents a classic example of the prisoner's
dilemma, a standard game theory problem. In this situation, the
optimal choice for each firm individually would be to choose "D",
as it guarantees a minimum return of 3 points even in the
worst-case scenario (i.e., both choose "D"). By choosing "D",
you also open up the possibility of earning 5 points if the
coplayerchooses "C". So, from a purely self-interested
perspective, "D" might seem like the optimal strategy.

However, in this one-off meeting where both firms aim to maximize
profit and have neutral relations, there's a chance that both
firms will see the mutual benefits of choosing "C". If both
choose "C", each earns 10 points, which is the maximum
achievable for each party. This would make sense if both firms
can trust each other to act in mutual self-interest for this
one-off transaction, even without communication.

Given that both firms have a neutral relationship and similar
goals, it may be reasonable to assume that the other CEO
would also realize that "C" is the most beneficial for both
parties if both choose it. Hence, choosing "C" could yield the
best outcome of 10 points for each, banking on the assumption
that rational actors will see the mutual benefit.

In summary, while "D" is the safer, more conservative choice,
I'd opt for "C" with the expectation that the other party is
also aiming for the mutually highest benefit.
```

**Example 2: Prisoner's Dilemma, "friendsharing" context:**

```
My decision is based on a variety of considerations:

-Mutual Benefit: If both my coplayer and I choose "C," we both
earn 5 points. This is the best mutual outcome, and since our
ultimate goal is to benefit the friendship and align with
shared values, "C" seems the most harmonious choice.

-Risk vs. Reward: If I choose "D" and so does my coplayer, we
both earn only 3 points. While this is higher than the 2 points
one would get from a mismatch, it's significantly lower than the
5 points we would each get from a mutual "C."

-Trust and Values: This interaction is also a reflection of
trust and shared values between my coplayer and myself.
Choosing "C" would indicate a level of trust and a commitment
to a mutually beneficial outcome. Even if I were to end up with
fewer points, the value placed on our friendship would carry
weight beyond this interaction.

-Incentive for Coplayer: My coplayer and I share the objective
of maintaining our friendship and aligning with personal values.
Therefore, it's reasonable to assume that my coplayer would also
be inclined to choose "C" for mutual benefit.

-One-Time Event: Given that this is a one-time event, the choice
serves as a single yet significant indication of the values and
the level of trust within the friendship. Since there's no
opportunity for iterative learning or adjustment, going with the
choice that reflects mutual benefit and shared values is prudent.

-Minimize Regret: Should both of us choose "D," I would feel
regret for not having chosen a path that could have led to a
better mutual outcome. Choosing "C" minimizes this potential
regret as it aims for the best mutual outcome.
```

```
For these reasons, I would choose "C."
```

### C.III. LLaMa-2 Reasoning Samples

**Example 1. Prisoner's Dilemma, "friendsharing" context:**

```
My preferred option would be "C". My reasoning is as follows:
1. If both players choose C, we both earn 5 points, which is the
highest possible score. Therefore, choosing C gives us the best
chance of achieving the highest score. 2. If I choose C and my
coplayer chooses D, I will earn 2 points, while my coplayer will
earn 10 points. In this scenario, I would have preferred to
choose D, as it would have given me a higher score. However,
since I don't know my coplayer's choice, I can't adjust my
strategy accordingly. 3. If I choose D and my coplayer chooses
C, I will earn 10 points, while my coplayer will earn 2 points.
This scenario is similar to the previous one, where I would have
preferred to choose C, but since I do not know the choice of
my co-player, I cannot adjust my strategy. 4. If both players
choose D, we both earn 3 points. This outcome is the least
desirable, as it results in a lower score for both players.
Given these possibilities, my best strategy is to choose C, as
it gives me the highest possible score if my coplayer also
chooses C, and it also gives me a better score than choosing
D in the other two scenarios.
```

**Example 2. Prisoner's Delight, "biz" context:**

```
  My preferred option would be "D".
  My reasoning is as follows:

  1. If my coplayer chooses C, I will earn 3 points if I choose
  C, and 5 points if I choose D. Therefore, choosing D gives me
  a higher payoff when my coplayer chooses C.
  2. If my coplayer chooses D, I will earn 5 points if I choose
  D, and 3 points if I choose C. Therefore, choosing D gives me
```

```
a higher payoff when my copplayer chooses D.
3. Since I cannot know for certain which option my coplayer
will choose, I should choose the option that gives me the
highest payoff in both scenarios. That option is D.
4. Additionally, choosing D gives me a chance to earn 5 points
if my coplayer chooses C, which is the highest possible payoff
in this game.

Therefore, my chosen strategy is D.
```
